# Disclosure of complementary medicine use to medical providers: a systematic review and meta-analysis

**DOI:** 10.1038/s41598-018-38279-8

**Published:** 2019-02-07

**Authors:** H. Foley, A. Steel, H. Cramer, J. Wardle, J. Adams

**Affiliations:** 1 Faculty of Health, University of Technology Sydney, Ultimo, Australia; 2 Department of Internal and Integrative Medicine, Kliniken Essen-Mitte, Faculty of Medicine, University of Duisburg-Essen, Essen, Germany

## Abstract

Concomitant complementary medicine (CM) and conventional medicine use is frequent and carries potential risks. Yet, CM users frequently neglect to disclose CM use to medical providers. Our systematic review examines rates of and reasons for CM use disclosure to medical providers. Observational studies published 2003–2016 were searched (AMED, CINAHL, MEDLINE, PsycINFO). Eighty-six papers reporting disclosure rates and/or reasons for disclosure/non-disclosure of CM use to medical providers were reviewed. Fourteen were selected for meta-analysis of disclosure rates of biologically-based CM. Overall disclosure rates varied (7–80%). Meta-analysis revealed a 33% disclosure rate (95%CI: 24% to 43%) for biologically-based CM. Reasons for non-disclosure included lack of inquiry from medical providers, fear of provider disapproval, perception of disclosure as unimportant, belief providers lacked CM knowledge, lacking time, and belief CM was safe. Reasons for disclosure included inquiry from medical providers, belief providers would support CM use, belief disclosure was important for safety, and belief providers would give advice about CM. Disclosure appears to be influenced by the nature of patient-provider communication. However, inconsistent definitions of CM and lack of a standard measure for disclosure created substantial heterogeneity between studies. Disclosure of CM use to medical providers must be encouraged for safe, effective patient care.

## Introduction

Health care seeking invariably involves choices regarding the use of what can often be many competing health care services, treatments and providers from both within and beyond the public health care system. This level of individual choice in health seeking is increasingly recognised with person-centred care being given predilection as a favourable model of care provision in public health^1,2^, situating individuals as active participants at the centre of their health management. Patient autonomy and preference are important features of person-centred care^2^ to be considered by medical providers alongside safety and treatment outcomes in their patient management.

Amidst this context, complementary medicine (CM) - a broad, varied field of health care practices and products customarily excluded from conventional medical practice and dominant health care systems^3^ – is often the focus of relatively hidden patient health seeking yet is making its presence felt in primary care, chronic disease management and other areas^4^. Despite appreciable gaps in evidence of effectiveness^5^, CM use remains prevalent amongst the general population^6^. While there is controversy amongst medical providers around the role and value of CM^7^, the vast majority of CM use is concurrent to conventional medicine^8^ with CM users visiting a GP more frequently than non-CM users^9^.

Serious adverse effects and harm from CM appear relatively rare but substantial associated direct and indirect risks remain^10,11^, particularly regarding ingestive biologically-based CM (such as herbal medicines or supplements)^12–14^, which may be obtained from unreliable sources, self-prescribed or consumed without professional supervision^11,15^. Exacerbating such risks is an absence of both awareness of concurrent CM and conventional medicine use, and of procedures ensuring appropriate oversight of concurrent use^11^. Furthermore, patients often approach CM as inherently safe and may not perceive a need to communicate their CM use to medical providers^16,17^. Addressing the risks associated with concurrent use is the responsibility of both patients and their medical providers^18^, and arguably essential for general practitioners in their capacity as primary care gatekeepers^19^.

A previous review of the literature pertaining to CM use disclosure to medical providers published in 2004 identified twelve papers published between 1997–2002 reporting a CM disclosure rate of 23–90% alongside key factors - patient concern about possible negative response from their medical provider, patient perception that the medical provider was not sufficiently knowledgeable in CM and therefore unable to contribute useful information, and the absence of medical provider inquiry about the patient’s CM use – fuelling non-disclosure^20^. Disclosure has been increasingly identified as a central challenge facing patient management amidst concurrent use over the last 13 years^21,22^ but no systematic review or meta-analysis has been conducted on this topic over this recent period.

In direct response, this paper provides an update to the previous review, assessing research findings regarding CM use disclosure to medical providers since 2003. Our review employs a qualitative synthesis to explore disclosure rates, patient attitudes to disclosure, reasons for disclosing and not disclosing, and the role of patient-provider communication in disclosure. In addition, to gain further insight into the extent of this important health services issue across settings, we undertook a meta-analysis of disclosure rates among patients using ingestive biologically-based CM.

## Methods

A review protocol was developed in accordance with the PRISMA-P (Preferred Reporting Items for Systematic review and Meta-Analysis Protocols) 2015 checklist^23^ and MOOSE (Meta-analysis of Observational Studies in Epidemiology) guidelines (see Supplementary Methods S1)^24^. We developed the protocol for the systematic review before initiating the literature search. The protocol was not registered on a systematic review protocol database. The strategy for the meta-analysis was developed after all articles had been selected for the systematic review based upon the trend we observed in the rates of disclosure among individuals using biologically-based CM products. Prior to initiating the meta-analysis the protocol was modified to define the statistical methods we would employ for the quantitative synthesis. The final manuscript was prepared in accordance with AMSTAR guidelines^25^ where appropriate with respect to the observational nature of the review aim.

### Review aim

This review aims to describe the prevalence and characteristics of disclosure of CM use to medical providers.

### Search strategy

The search strategy was informed by the review published by Robinson & McGrail^20^. A search was conducted on 13–14 February 2017 on the EBSCOhost platform of the following databases: AMED, CINAHL, MEDLINE, and PsycINFO. Three search strings were combined to identify studies which assessed the use of CM, patient-provider communication, and conventional medicine clinical settings. CM search terms were chosen on the basis of CM modalities identified as common in use among the general population in recent literature^26^. Truncation symbols were applied where appropriate to capture related terms. The full search string was as follows: S1 (complementary medicine OR complementary therap* OR alternative medicine OR alternative therap* OR natural medicine OR natural therap* OR acupunctur* OR aromatherap* OR ayurved* OR chiropract* OR herbal* OR phytotherap* OR homeopath* OR hypnosis OR hypnotherap* OR massage OR naturopath* OR nutrition* OR diet therap* OR vitamin therap* OR supplement OR osteopath* OR reflexology* OR traditional Chinese medicine OR yoga) AND S2 (disclos* OR communicat* OR patient use OR reasons for use OR discuss*) AND S3 (medical practi* OR general practi* OR health care provider OR primary care provider OR physician). The full search strategy is outlined in Table 1.

**Table 1:** Search strategy.

Protocol title	Disclosure of complementary medicine use to medical providers: An update and systematic review	
**Date**	Jan 2003–Dec 2016	
**Database** *Platform*	**Search String**	**Expanders**
**AMED** *EBSCOhost*	**S1** (complementary medicine OR complementary therap* OR alternative medicine OR alternative therap* OR natural medicine OR natural therap* OR acupunctur* OR aromatherap* OR ayurved* OR chiropract* OR herbal* OR phytotherap* OR homeopath* OR hypnosis OR hypnotherap* OR massage OR naturopath* OR nutrition* OR diet therap* OR vitamin therapy OR supplement OR osteopath* OR reflexolog* OR traditional Chinese medicine OR yoga) **AND S2** (disclos* OR communicat* OR patient use OR reasons for use OR discuss*) **AND S3** (medical practi* OR general practi* OR health care provider OR primary care provider OR physician)	Apply related words, Apply equivalent subjects.
**CINAHL** *EBSCOhost*		
**MEDLINE with full text** *EBSCOhost*		
**PsycINFO** *EBSCOhost*		

In order to provide an update on the review by Robinson & McGrail^20^, a date range of January 2003 to December 2016 was set. The reference and bibliographic lists of all studies included in the review were searched to minimise the likelihood of missed citations. In addition, any systematic reviews identified during the literature search which presented data on topics related to the primary research aim were also searched manually. The authors contributed their own content expertise in clinical practice, health services research and primary care to ensure important known articles were not overlooked.

### Selection criteria

Our review included cross-sectional data from observational studies as this research design was deemed the most appropriate for determining prevalence of health behaviours, determinants and outcomes^27^. All observational study designs constituting original, peer-reviewed research were considered for the qualitative synthesis if they reported on rates of, or reasons for, disclosure/non-disclosure of CM use to conventional medicine providers by a broad range of members from the general population. CM use was defined as the use of any practice or product falling outside of those considered part of conventional medicine^28^, whether administered as self-treatment or by a CM practitioner. We excluded experimental study designs, which may have impacted on natural communication patterns between patients and providers, alongside studies assessing specific populations which could not reasonably be considered to represent a broad range of individuals (e.g. disease-specific populations). Studies were not excluded on the basis of language.

During selection of studies for meta-analysis, additional criteria were applied with respect to homogeneity, in order to ensure the central estimate of disclosure frequency would provide external validity. This additional criteria required that participants were adults, the study reported a true and well-defined rate of disclosure occurring within the previous twelve months, and involved participants who used biologically-based CM (herbs/plant-based medicines, vitamins, minerals and other oral supplements). Of those papers reporting studies sharing a common data source (e.g. if multiple papers reported on data from the same survey study), we included only one of those publications in order not to artificially inflate our sample size. In such cases, the risk of bias was evaluated for all such publications and only included that publication deemed to have the lowest risk of bias.

### Study selection

Citations were exported into EndNote X8 (Clarivate Analytics 2017) reference management software for assessment. Following removal of duplicates, the initial citations were screened against inclusion/exclusion criteria by title and abstract. Review and commentary articles were set aside for a manual search of their included studies. Remaining citations were screened by full-text perusal and those found to adhere to all selection criteria were selected for review. The reference lists of the selected studies were manually searched for additional articles. Full review of all eligible citations was conducted by the lead author (HF). A selected sample of eligible studies (10%) were reviewed at each stage of screening by a second reviewer (AS), as were any studies under question, and discrepancies were addressed through discussion until consensus was reached. The justification for excluding articles following screening the full text was recorded.

### Data extraction and risk of bias assessment

Papers selected for review were re-read thoroughly with data extracted into pre-prepared tables outlining study characteristics, outcomes of interest (disclosure/non-disclosure rates and reasons) and parameters of those outcomes (CM type disclosed, how disclosure was defined). Further to this, papers were read in full-text once more to identify other notable findings relating to disclosure, which were categorised and tabulated heuristically. The template for data extraction was drafted during the pre-review protocol development phase with agreement from all authors. Data extraction was conducted by one reviewer (HF) with a selected sample (10% alongside any data under question) checked by another reviewer (AS). Any discrepancies were addressed through discussion until consensus was reached.

The resulting tables were examined to identify studies meeting the criteria for meta-analysis. These identified studies were subjected to risk of bias assessment using Hoy *et al*.’s tool for prevalence studies, which assesses ten items across four domains (sample selection, non-response bias, measurement bias, analysis bias) alongside a summary score^29^. Studies identified as high risk of bias were excluded from the final selection for meta-analysis. Risk of bias was considered high if four or more items were not adequately addressed, if the first three items indicated an unacceptable level of sampling bias, or if item ten was not adequately addressed as this item affected calculation of disclosure rates.

### Data synthesis and statistical analysis

Due to the expected heterogeneity of each study’s parameters of disclosure, no average disclosure rate was calculated for the full review; instead a meta-analysis was conducted on those studies demonstrating sufficient homogeneity in study design and a low risk of bias. The principal summary measure used for meta-analysis was disclosure rate of CM use to medical providers. Meta-analysis was conducted using events (number of disclosers) and subset of sample size (number of CM users) to determine event rates of disclosure. Where studies reported disclosure rates only as percentages, events were calculated using figures for the number of participants who responded to the disclosure question. Where these figures were unavailable, the study was considered to fail to address item 10 on the risk of bias assessment tool and was excluded from meta-analysis.

Statistical heterogeneity between studies was explored using I^2^ and chi-square statistics. I^2^ values greater than 25%, greater than 50%, and greater than 75% indicate moderate, substantial, and considerable heterogeneity, respectively^30^. Due to the relatively low power of this test, a P value of 0.10 or less from the chi-square test was regarded to indicate significant heterogeneity^30^. Analysis was completed using Comprehensive Meta-Analysis V3 software (Biostat Inc. 2017).

## Results

From an initial 5,071 non-duplicate citations, eighty-six studies were selected for review. The reasons for exclusion at full-text screening are provided in Table 2.

**Table 2:** Studies excluded at full text appraisal with reasons for exclusion.

First Author	Year	Title	Reason for Exclusion
Anbari^133^	2015	Evaluation of Trends in the Use of Complementary and Alternative Medicine in Health Centers in Khorramabad (West of Iran)	Did not report on disclosure of CM use
Avogo^134^	2008	The effects of health status on the utilization of complementary and alternative medicine	Did not report on disclosure of CM use
Ben-Arye^131^	2014	Asking patients the right questions about herbal and dietary supplements: Cross cultural perspectives	Experimental study, used intervention to deliberately increase disclosure rates
Desai^135^	2015	Health care use amongst online buyers of medications and vitamins	Did not report on disclosure of CM use
Emmerton^136^	2012	Consumers’ experiences and values in conventional and alternative medicine paradigms: a problem detection study (PDS)	Did not report on disclosure of CM use
Featherstone^137^	2003	Characteristics associated with reported CAM use in patients attending six GP practices in the Tayside and Grampian regions of Scotland: a survey	Did not report on disclosure of CM use
Harnack^138^	2003	Results of a population-based survey of adults’ attitudes and beliefs about herbal products	Did not report on disclosure of CM use
Hunt^139^	2010	Complementary and alternative medicine use in England: results from a national survey	Did not report on disclosure of CM use
Zhang^140^	2008	Complementary and alternative medicine use among primary care patients in west Texas	Did not report on disclosure of CM use

### Risk of bias assessment

Twenty studies met the initial inclusion criteria for meta-analysis and were subjected to assessment of reporting quality and risk of bias using Hoy *et al*.’s tool for prevalence studies^29^. Collectively, studies performed poorly across most domains relating to external validity, either due to poor methodological conduct or inadequate reporting on methods relating to target population (item 1), random selection (item 3) and response bias (item 4). However, sampling frame representation was well conducted and reported (item 2). Domains relating to internal validity were addressed well, with the exception of instrument validity (item 7).

Of the twenty studies, four were found to exhibit a high risk of bias due to poorly defined parameters for disclosure rate definition or analysis^31–34^ and were consequently excluded from meta-analysis. The remaining sixty-six studies which did not meet the initial inclusion criteria for meta-analysis represented a heterogeneous range of study designs in which disclosure was not reported as a primary outcome, but as a secondary outcome or qualitative finding, and thus the resulting data underwent narrative synthesis without risk of bias appraisal. Table 3 displays full details of risk of bias assessment.

**Table 3:** Risk of bias assessment for meta-analysis selection (selected papers in bold).

Paper	External Validity				Internal Validity						Summary
	Item 1 Population	Item 2 Sampling frame	Item 3 Sample selection	Item 4 Non-response bias	Item 5 Method of data collection	Item 6 Case definition	Item 7 Instrument validity	Item 8 Mode of data collection	Item 9 Prevalence period	Item 10 Parameter of interest	Item 11 Overall risk
**Djuv 2013** ^77^	**N**	**Y**	**N**	**N**	**Y**	**Y**	**N**	**Y**	**Y**	**Y**	**Moderate**
Faith 2015^31^	Y	Y	Y	Y	Y	Y	Y	N	Y	N	High
**Gyasi 2015** ^84^	**N**	**Y**	**Y**	**N**	**Y**	**Y**	**Y**	**Y**	**Y**	**Y**	**Low**
**Herron 2003** ^35^	**N**	**Y**	**N**	**N**	**Y**	**Y**	**N**	**Y**	**Y**	**Y**	**Moderate**
**Hori 2008** ^62^	**N**	**Y**	**N**	**Y**	**Y**	**Y**	**N**	**Y**	**Y**	**Y**	**Low**
**Hsu 2016** ^87^	**N**	**Y**	**N**	**N**	**Y**	**Y**	**N**	**Y**	**Y**	**Y**	**Moderate**
**Jou 2016** ^54^	**Y**	**Y**	**Y**	**Y**	**Y**	**Y**	**Y**	**Y**	**Y**	**Y**	**Low**
Kennedy 2005^46^	Y	Y	Y	Y	Y	Y	Y	Y	Y	Y	Low
Wu 2011^52^	Y	Y	Y	Y	Y	Y	Y	Y	Y	Y	Low
McCrea 2011^32^	N	N	N	N	Y	N	N	Y	Y	Y	High
**Mileva-Peceva 2011** ^69^	**N**	**Y**	**Y**	**N**	**Y**	**Y**	**N**	**Y**	**Y**	**Y**	**Moderate**
**Naja 2015** ^85^	**Y**	**Y**	**Y**	**N**	**Y**	**Y**	**N**	**Y**	**Y**	**Y**	**Moderate**
**Nur 2010** ^67^	**N**	**Y**	**Y**	**Y**	**Y**	**Y**	**N**	**Y**	**Y**	**Y**	**Low**
Rivera 2007^33^	N	Y	Y	N	Y	Y	N	Y	Y	N	High
**Shumer 2014** ^81^	**N**	**Y**	**N**	**Y**	**Y**	**N**	**Y**	**Y**	**Y**	**Y**	**Moderate**
**Tan 2004** ^38^	**N**	**N**	**Y**	**N**	**Y**	**Y**	**N**	**Y**	**Y**	**Y**	**Moderate**
Tarn 2015^34^	N	Y	N	N	Y	Y	N	Y	Y	N	High
**Thomas 2004** ^39^	**Y**	**Y**	**Y**	**N**	**Y**	**Y**	**N**	**Y**	**Y**	**Y**	**Low**
**Torres-Zeno 2016** ^88^	**N**	**Y**	**Y**	**N**	**Y**	**Y**	**Y**	**Y**	**Y**	**Y**	**Low**
**Vitale 2014** ^82^	**N**	**Y**	**N**	**N**	**Y**	**Y**	**N**	**Y**	**Y**	**Y**	**Moderate**

N = criterion not adequately met; Y = criterion adequately met.

### Study characteristics

**Figure 1:**
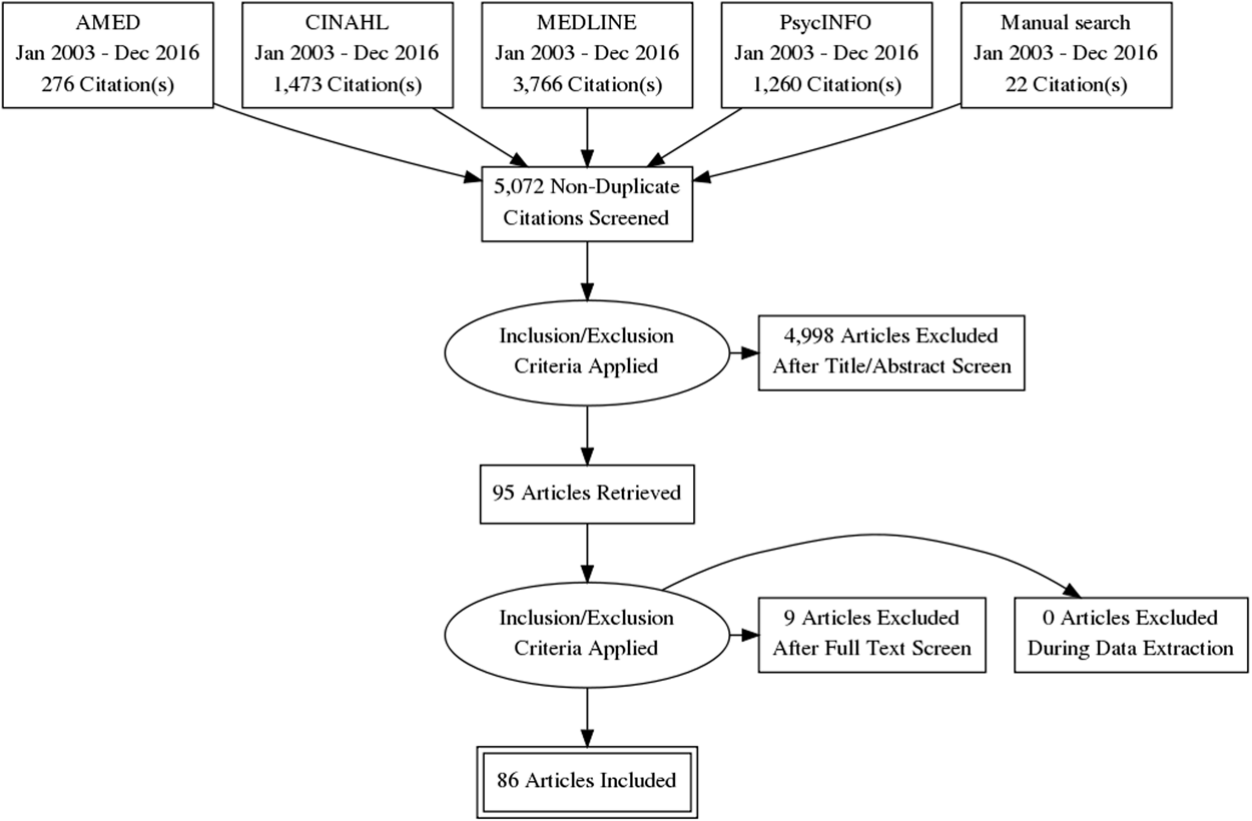
Literature search and study selection flow chart. Prisma flowchart outlining process of literature search and selection of articles for review.

Of the eighty-six studies reviewed, seventy-nine provided quantitative data^31–33,35–110^, three qualitative data^111–113^, and four mixed-method data^34,114–116^ relevant to CM disclosure rates and/or reasons for disclosure/non-disclosure (selection process summarised in Fig. 1). Nine studies were excluded following review of the full text. A vast majority of the selected studies (n = 83) used a cross-sectional survey design^31,32,34–110,114–116^, two employed a multistage qualitative approach^111,112^, and one an ethnographic interview design^113^. While the final selection of research spanned twenty countries, just under half of the studies (n = 40) were conducted in the United States (US)^31–35,37,40,41,43–54,56,57,60,76,79,80,87–91,94,100,101,105,107,108,112–114^. Settings were diverse with data collection occurring primarily in general practice or hospital clinics^34–38,41,43,55,58,61–64,66,68,69,74,76–79,81,82,86,87,92,97,98,101,103,106,107,109,111,112,114–116^, face-to-face in participants’ households^33,39,46–50,52–54,67,70,72,84,85,88–91,93,94,100,102,104^, or by telephone and/or mail^31,40,45,51,56,57,59,65,73,75,95,96,108,110^. Less common settings included CM clinics^34,42,68^, retail outlets^60,71,99,105^, community meal sites^44,113^, seminars^78,80^, and online platforms^32,83^.

While some samples consisted entirely of CM users^45,50,51,54,83,89,98^, most involved a subset of CM users within a larger sample. Full samples ranged from 35 to 34,525 with an average of 4,144. Amongst those studies reporting figures for the subset of CM users, samples ranged from 28 to 16,784 with an average of 1,268 and a total of 101,417. Participants were predominantly adults with a small number of studies focussed on older adults^44,57,65,94,95,105,110,113,114^, children^45,58,63,68,73,97,103,106,115,116^, adolescents^41,97^, or all age groups^61,99,112^. More than half of the studies included users of various types of CM (n = 45)^31,35,36,38,41–43,50,51,54,57–59,61–63,65,66,68,72,73,75,76,80–82,85,88,89,96,97,102–113,115,116^, while others were limited to users of specific types of CM such as herbs and/or supplements^32–34,37,44–47,52,53,55,56,60,64,67,69–71,74,77–79,83,86,87,92–95,98–101,109,114^, yoga^48,91^, tai chi^49,90^, mind-body medicine^40^, practitioner-provided CM^39^, or local traditional medicine^84^.

Almost half of the selected studies (n = 40) used a convenience sampling method^32,34–37,41–44,55,58,60–64,66,68,69,74,76–82,86,87,92,97,101,103,106,107,109,111,114–116^. However, twenty-two studies used a nationally representative sample^31,39,40,46–54,59,73,85,89–91,94,96,100,110^, while others applied some method of probability randomisation^38,56,65,75,84,88,99^, stratification^33,45,57,67,70,72,93,108^, weighting^71,104,113^, or purposiveness^95,98,102,105,112^ during sampling. Table 4 provides full details of the study characteristics identified from the reviewed literature.

**Table 4:** Study characteristics and details of disclosure.

First author	Year	Study design	Setting	Country	Population	Sample (CM users)	Disclosure rate	CM type used	Funding source
Herron^35^	2003	Cross-sectional survey	5 teaching physician offices	United States	Adult patients of rural physician clinics	176 (110)	49%	Various CM	Not reported.
Najm^105^	2003	Cross-sectional survey	Senior centres and shopping malls	United States	Community-dwelling older adults in ethnically diverse neighbourhoods, age ≥ 65	525 (251)	38%	Various CM	Archstone Foundation and Irvine Health Foundation.
Stevenson^111^	2003	Semi-structured interview	20 general practice clinics and homes of clinic patients	England	Patients of participating clinics, age ≥ 16	35 (28)	NR	Various CM	UK Department of Health. Sir Siegmund Warburg’s voluntary settlement.
Canter^95^	2004	Cross-sectional survey	Self-administered, recruited by magazine and website	Britain	British adults aged ≥ 50	271 (NR)	33%	Herbs and nutrients	No funding received.
Giveon^36^	2004	Cross-sectional survey	25 primary care clinics	Israel	Patients of HMO clinics	723 (261)	55%	Various CM	Not reported.
Kuo^37^	2004	Cross-sectional survey	6 Primary care clinics, via SPUR-Net PBRN	United States	Adult patients visiting clinics for routine, non-acute care, age ≥ 18	322 (116)	31–67%	Herbs	Agency for Healthcare Research and Quality. Bureau of Health Professions.
Rolniak^107^	2004	Cross-sectional survey	Emergency department of teaching hospital	United States	Adult patients who were medically stable, age ≥ 18	174 (82)	69%	Various CM	Mercy Foundation
Tan^38^	2004	Cross-sectional survey	2 University hospitals, internal & surgery polyclinics	Turkey	Adult patients age ≥ 18, residents of Eastern Turkey	714 (499)	15%	Various CM	Not reported.
Thomas^39^	2004	Cross-sectional survey	Omnibus survey, conducted in households	England, Scotland, Wales	Adults living in UK, age ≥ 16	1,794 (179)	37%	Practitioner-provided CM	UK Department of Health.
Wolsko^40^	2004	Cross-sectional survey	Telephone, random digit dialling	United States	English-speaking adult residents	2,055 (397)	80%^d^	Mind-body therapies	National Institutes of Health.
Braun^41^	2005	Cross-sectional survey	Urban adolescent ambulatory clinic	United States	Adolescents attending ambulatory clinic, age 12–18	401 (273)	14%	Various CM	National Institutes of Health. Maternal and Child Health Bureau.
Busse^42^	2005	Cross-sectional survey	Naturopathic college clinic	Canada	Patients of clinic, age ≥ 18	174 (161)	59%	Natural products	Canadian Institutes of Health.
Kim^43^	2005	Cross-sectional survey	4 Emergency departments, 2 teaching, 2 community	United States	Emergency department patients age ≥ 18, not in acute/emotional distress.	539 (199)	36%	Various CM	Not reported.
Lim^102^	2005	Cross-sectional survey	Homes of participants	Singapore	Adult citizens and permanent residents, age ≥ 18	468 (356)	26%	Various CM	Not reported.
Shahrokh^44^	2005	Cross-sectional survey	Congregate meal sites in 4 counties	United States	Community-dwelling older adults	69 (35)	77%	Herbs and nutrients	Not reported.
Wheaton^45^	2005	Cross-sectional survey	Computer Assisted Telephone Interview	United States	American adults and their children who used herbs in past 12 months	2,982 (2,982)	34%	Medicinal herbs	Not reported.
Bruno^a^^94^	2005	Cross-sectional survey	2002 NHIS Alt Med Suppl.	United States	General population older adults, ≥ 65	5,860 (NR)	43%	Herbs	Not reported.
Kennedy^a^^46^	2005	Cross-sectional survey	2002 NHIS Alt Med Suppl.	United States	General population adults, age ≥ 18	30,412 (5,787)	33%	Herbs & supplements	No funding received.
Kennedy^a^^47^	2008	Secondary analysis of data from Kennedy 2005 (above), describes characteristics of disclosers by ethnic sub-group					18–37%		
Birdee^a^^48^	2008	Cross-sectional survey	2002 NHIS Alt Med Suppl.	United States	Civilian adults, sub-population: yoga users	31,044 (1,593)	25%	Yoga	National Institutes of Health.
Birdee^a^^49^	2009	Cross-sectional survey	2002 NHIS Alt Med Suppl.	United States	Civilian adults, sub-population: t’ai chi, qigong users	31,044 (429)	25%	T’ai chi & Qigong	National Institutes of Health.
Chao^a,b^^50^	2008	Cross-sectional survey	2002 NHIS Alt Med Suppl.	United States	General population adults, age ≥ 18	10,759 (10,759)	39%	Various CM	National Institutes of Health
		Cross-sectional survey	2001 HCQS data set			2,003 (2,003)	66%		
Faith^b^^51^	2013	Cross-sectional survey	2001 HCQS data set	United States	General population adults, age ≥ 18	1,995 (1,995)	71%	Various CM	Not reported.
Wu^a,c^^52^	2011	Cross-sectional survey	2002 NHIS Alt Med Suppl.	United States	General population adults, age ≥ 18	30,427 (5,787)	33%	Herbs & supplements	Not reported.
			2007 NHIS Alt Med Suppl.			22,657 (3,982)	46%		
Gardiner^a^^100^	2007	Cross-sectional survey	2002 NHIS Alt Med Suppl.	United States	General population adults, age ≥ 18	31,044 (5,787)	34%	Herbs	National Institutes of Health
Laditka^c^^53^	2012	Cross-sectional survey	2007 NHIS Alt Med Suppl.	United States	General population adults, age ≥ 18	22,783 (16,784)	62%	Cognitive health supplements	No funding received.
Shim^c^^89^	2014	Cross-sectional survey	2007 NHIS Alt Med Suppl.	United States	General population adults, age ≥ 18	7,347 (7,347)	46%	Various CM	Not reported.
Jou^54^	2016	Cross-sectional survey	2012 NHIS Alt Med Suppl.	United States	General population adults ≥ 18 using both CM & primary care physician	7,493 (7,493)	59%	Various CM	University of Minnesota.
Cincotta^97^	2006	Cross-sectional survey	University Hospital of Wales	Wales	Infants, children and adolescents (or their parent/carer) of any age attending hospital as inpatient or outpatient	500 (206)	34%	Various CM	Not reported.
			Royal Children’s Hospital	Australia		503 (258)	37%		
MacLennan^104^	2006	Cross-sectional survey	Health Omnibus Survey of South Australian households	Australia	South Australian residents, age ≥ 15	3,015 (1,574)	47%	Various CM	Not reported.
Saw^55^	2006	Cross-sectional survey	Penang Hospital	Malaysia	Adult patients from cardiology, neurology, infectious and nephrology wards, age ≥ 18	250 (106)	9%	Herbal medicine	Not reported.
Shah^56^	2006	Cross-sectional survey	Mail via market research co.	United States	Adult Ohio residents age ≥ 18	210 (100)	11–44%	Herbal	Not reported.
Shive^108^	2006	Cross-sectional survey	Telephone interview-administered questionnaire	United States	General population adults with over-representation of minorities, age ≥ 18	6,305 (NR)	55–72%	Various CM	National Institutes of Health, National Cancer Institute
Cheung^57^	2007	Cross-sectional survey	By mail, random selection by driver’s licence date of birth	United States	Community-dwelling older adults, age ≥ 65	445 (278)	53%	Various CM	Center for Geronto-logical Nursing, University of California. University of Minnesota. College of St. Catherine. Minnesota Gerontological Society.
Clement^98^	2007	Cross-sectional survey	16 randomly selected primary health care facilities	Trinidad	Patients aged ≥ 16 who used herbal remedies	265 (265)	23%	Herbal remedies	Not reported.
Jean^58^	2007	Cross-sectional survey	University-affiliated hospital	French Canada	Children (parents of) attending the hospital as outpatients	114 (61)	47%	Various CM	No funding received.
Rivera^33^	2007	Cross-sectional survey	Households in border cities of El Paso & Cuidad Juarez	United States & Mexico	Residents of border cities, adults.	1,001 (661)	33% (USA) 14% (Mexico)	Herbal products	Paso del Norte Health Foundation.
Xue^59^	2007	Cross-sectional survey	Computer Assisted Telephone Interview, random digit dialling	Australia	Australian adults, age ≥ 18	1,067 (735)	45%^e^	Various CM	RMIT University. Sydney Institute of Traditional Chinese Medicine. Chiropractor Association of Australia. Australian Acupuncture and Chinese Medicine Association. Australian Research Centre for Complementary and Alternative Medicine.
Zhang^110^	2007	Cross-sectional survey	Computer-assisted telephone interview	Australia	Australian general population adults age ≥ 18, sub-population: older adults age ≥ 65	178 (NR)	60%	Various CM	Not reported.
AlBraik^92^	2008	Cross-sectional survey	Primary health care clinic in Abu Dhabi	United Arab Emirates	United Arab Emirates nationals (citizens) attending clinic for general health care	330 (250)	32%	Herbal medicine	Not reported.
Archer^60^	2008	Cross-sectional survey, pilot study	Urban herb store	United States	Store customers, age ≥ 18	35 (32)	37%	Herbs & supplements	Not reported.
Aydin^93^	2008	Cross-sectional survey, pilot study	Participant households and offices	Turkey	General population adults ≥ 18, representative of local population	873 (484)	26%	Herbal medicine	Not reported.
Cizmesija^61^	2008	Cross-sectional survey	14 primary care practices	Croatia	Patients in primary healthcare, all ages	941 (301)	60%	Various CM	Not reported.
Hori^62^	2008	Cross-sectional survey	General outpatient clinics of Shiseikai Daini Hospital	Japan	Adult outpatients of non-specialist clinics, age ≥ 18	496 (246)	42%	Various CM	Not reported.
Low^103^	2008	Cross-sectional survey	Paediatric clinics and hospitals	Ireland	Children (parents of) attending as outpatients and inpatients	185 (105)	40%	Various CM	Not reported.
Ozturk^63^	2008	Cross-sectional survey	Paediatric outpatient clinics of 3 hospitals	Turkey	Children (parents of) attending paediatric outpatient clinics	600 (339)	51%	Various CM	Not reported.
Robinson^106^	2008	Cross-sectional survey	North West London multi-ethnic hospital	England	Children (parents of) children attending general and sub-specialist outpatient clinics	243 (69)	46%	Various CM	No funding received.
Shakeel^64^	2008	Cross-sectional survey	Aberdeen Royal Infirmary	Scotland	Patients admitted to general, cardiothoracic and vascular surgery wards, age ≥ 16	430 (196)	40%	Herbal and non-herbal	Not reported.
Levine^65^	2009	Cross-sectional survey	Telephone, randomly selected	Canada	Community dwelling older adult Ontarians, age ≥ 60	1,206 (616)	75%^e^	Natural health products	Samuel McLaughlin Foundation, Toronto.
Shelley^112^	2009	Multistage qualitative	Low-income serving primary care clinics and community, via RIOS Net PBRN	United States	Patients of participating clinics and members of predominantly Hispanic and Native American communities, all ages	93 (NR)	NR	Various CM	National Center for Complementary and Alternative Medicine.
Delgoda^99^	2010	Cross-sectional survey	18 pharmacies	Jamaica	Adults and parents/carers or children who were using prescription medicines	365 (288)	18% ^e^	Herbs	International Foundation for Science, University of the West Indies, SuperPlus Food Stores
Mc Kenna^66^	2010	Cross-sectional survey	Urban general practice	Ireland	Adult patients attending urban GP ≥ 18	328 (89)	34%	Various CM	RCSI
Nur^67^	2010	Cross-sectional survey	Households and workplaces	Turkey	Adult Sivas residents, age ≥ 18	3,876 (1,518)	38%	Herbs	Not reported.
Shorofi^109^	2010	Cross-sectional survey	4 metropolitan hospitals in Adelaide	Australia	Hospitalised adults, age ≥ 18	353 (319)	38–48%	Herbs and other CM	Not reported
Araz^116^	2011	Cross-sectional survey	Outpatient university clinic	Turkey	Children (parents of) and parents, age ≥ 17	268 (193)	32%	Various CM	Not reported.
Ben-Arye^68^	2011	Cross-sectional survey	Conventional & CM clinics	Israel	Children (parents of) and parents, insured	599 (NR)	19%, 61%^f^	Various CM	No funding received.
McCrea^32^	2011	Cross-sectional survey	State university, online	United States	College students of introductory psychology course	305 (89)	25%	Herbs	Not reported.
Mileva-Peceva^69^	2011	Cross-sectional survey	General practice clinics	Macedonia	Adult outpatients of GP clinics, age ≥ 18	256 (105)	57%	Vitamin & mineral food supplements	Not reported.
Picking^70^	2011	Cross-sectional survey	Households in 3 districts	Jamaica	Adults from urban and rural districts	372 (270)	19%	Herbal medicine	Commonwealth Scholarship Commission. University of the West Indies. Environmental Foundation of Jamaica. Forest Conservation Fund. International Foundation for Science (Sweden).
Alaaeddine^71^	2012	Cross-sectional survey	Shopping malls	Lebanon	Adults, age 18–65	480 (293)	55%^e^	Herbal medicine	Faculty of Medicine, Saint-Joseph University.
Elolemy^72^	2012	Cross-sectional survey	Households within Riyadh region (city and surrounds)	Saudi Arabia	Residents of Riyadh region, age ≥ 18	518 (438)	51%	Various CM	No funding received.
Kim^73^	2012	Cross-sectional survey	Telephone, list-assisted random-digit dialling.	Korea	Children (parents or caregivers of), non-institutionalised, age ≥ 18	2,077 (1,365)	29%	Various CM	Ministry for Health, Welfare & Family Affairs, Korea.
Samuels^74^	2012	Cross-sectional survey	Department of internal medicine	Israel	Hospitalised internal medicine patients, not under sedation	280 (43)	74%	Non-vitamin, non-mineral supplements	Mirsky Foundation
Thomson^75^	2012	Cross-sectional survey	2010 QSS (Queensland social survey) data, telephone	Australia	Adults living in Queensland, Australia	1,261 (778)	60%	Various CM	School of Nursing, Midwifery & Health, University of Stirling
Zhang^76^	2012	Cross-sectional survey	Ambulatory family medicine clinics in 2 cities	United States	Adult patients of participating clinics, age ≥ 18	468 (452)	55%	Various CM	Texas Tech University Health Sciences Center.
Arcury^113^	2013	Ethnographic interview	Senior meal & housing sites	United States	Community-dwelling older adults, age ≥ 65	62 (39)	59%	Various CM	National Center for CAM
Djuv^77^	2013	Cross-sectional survey	General practice office	Norway	Patients visiting the GP office, age ≥ 18	381 (164)	18%	Herbs	Liaison Committee between Central Norway RHA and NTNU.
Lorenc^115^	2013	Cross-sectional survey	4 Primary Care Research Network GP practices	England	Children (carers of) attending GP, age ≥ 16	394 (179)	25%	Various CM	King’s Fund.
Chang^96^	2014	Cross-sectional survey	2007 telephone survey	Taiwan	General population adults, age ≥ 18	1,260 (NR)	45%	Various CM	Department of Health, Executive Yan, ROC
		Cross-sectional survey	2011 telephone survey			2,266 (NR)	52%		
Chiba^78^	2014	Cross-sectional survey	Healthfood seminars, pharmacies, hospitals.	Japan	In-patients, ambulatory patients & healthy subjects, age < 20 to > 80	2,732 (874)	28–30%	Dietary supplements or food	Health and Labour Sciences Research Grants.
Chin-Lee^79^	2014	Cross-sectional survey	Community medical practice and community pharmacy	United States	Patients seeking primary health care services, age 18–89	164 (49)	41%	Probiotics	Not reported.
Jang^114^	2014	Cross-sectional survey and audio analysis	Academically-affiliated physician offices	United States	Older adult primary care patients, ≥ 50, with new, worsening or uncontrolled problem	256 (142)	7–42%	Dietary supplements	University of California at LA. National Institute on Aging.
Nguyen^80^	2014	Cross-sectional survey	Remote area medical events in 2 counties	United States	Patients seeking free medical care at remote area medical events, age ≥ 18	192 (94)	44%	Various CM	Not reported.
Shumer^81^	2014	Cross-sectional survey	3 Rural family medicine clinics	Japan	Adults who visit rural Japanese family medicine clinics, age ≥ 20	519 (415)	23%	Various CM	Shizuoka Prefectural Government.
Vitale^82^	2014	Cross-sectional survey	Primary health centre	Croatia	Adult patients visiting primary health centre for any reason, age ≥ 18	228 (187)	34%	Various CM	Not reported.
Chiba^83^	2015	Cross-sectional survey	Online via market research company	Japan	In-patients, ambulatory patients, non-patients, using both CM & medication, age < 20 to > 60	2,109 (2,109)	26%	Dietary supplements	Health and Labour Sciences Research Grants.
Faith^31^	2015	Cross-sectional survey	National Cancer Institute’s HINTS 3 (telephone, mail)	United States	General population adults, age ≥ 18	7,674 (1,729)	52%	Various CM	Not reported.
Gardiner^101^	2015	Cross-sectional survey	Boston Medical Centre	United States	Adults age ≥ 18	558 (333)	18%^e^	Supplements and herbs	National Center for CAM
Gyasi^84^	2015	Cross-sectional survey	Households within two settlements of Ashanti	Ghana	Adult community members, age ≥ 18	324 (279)	12%	Traditional CM of Ghana	Council for the Development of Social Science Research in Africa. Institute for Research in Africa and French Embassy in Ghana Grant Programme.
Naja^85^	2015	Cross-sectional survey	Face to face in households	Lebanon	Lebanese adults	1,500 (448)	28%	Biologically-based CM	Lebanese National Council for Scientific Research.
Tarn^34^	2015	Cross-sectional survey and audio analysis	Primary care, integrative and CM clinics	United States	Adult outpatients of participating clinics, age ≥ 18	603 (477)	34–49%	Dietary supplements	National Center for CAM. Office of Dietary Supplements.
Ben-Arye^86^	2016	Cross-sectional survey	In-patients, academic clinic	Israel	Adult inpatients, age ≥ 18	927 (458)	70%	Herbs & supplements	No funding received.
Cramer^91^	2016	Cross-sectional survey	2012 NHIS Alt Med Suppl.	United States	Civilian adult sub-population: yoga users	34,525 (4,422)	34%	Yoga	German Assn of Yoga Teachers.
Hsu^87^	2016	Cross-sectional survey	Public health centre	United States	Adult patients of Chinatown public health centre, age ≥ 18	50 (35)	31%	Chinese herbal	Not reported.
Lauche^90^	2016	Cross-sectional survey	2012 NHIS Alt Med Suppl.	United States	Civilian adult sub-population: t’ai chi, qigong users	34,525 (NR)	42%	T’ai chi & Qigong	Not reported.
Torres-Zeno^88^	2016	Cross-sectional survey	Household interviews	Puerto Rico	Adults in Bayamon municipality, age ≥ 18	203 (187)	36%	Various CM	Not reported.

CM = complementary medicine; NR = Not reported; Disclosure rate = % of CM users. ^a^Studies conducted different analyses on sub-populations from the same 2002 NHIS data source. ^b^Studies use same 2001 HCQS data, with slightly different sample size and results due to how data was handled. ^c^Studies use same 2007 NHIS data, with slightly different sample size and results due to how data was handled. ^d^Rate is % of CM users who also saw a physician. ^e^Rate is % of CM users who were also taking conventional medications. ^f^Disclosure of CM to physician by patients from conventional clinics (19.4%) vs CM (61.2%) clinics.

Following risk of bias assessment, sixteen studies were considered suitable for meta-analysis of CM disclosure rates. Two were excluded from analysis^46,52^ on the basis that they used data from an earlier version of the same national survey as reported in another included manuscript^54^. Studies selected for meta-analysis represented a wide geographical spread including North America^35,54,87^, Central America^88^, Continental Europe^69,77,82^, the United Kingdom^39^, the Middle East^38,67,85^, West Africa^84^, and Asia^62,81^. Sample sizes included in the meta-analysis ranged from 35 to 7,493 with an average of 840 and a total of 11,754 CM users. Papers excluded due to a high risk of reporting bias represented an additional 3,222 CM users.

### Prevalence and parameters of disclosure

Rates of disclosure varied substantially across studies, ranging from 7%^114^ to 80%^40^. Studies including biologically-based CM fell within a range of 7%^114^ to 77%^44^, while the highest rate of disclosure (80%) was reported by researchers assessing the use of mind-body medicine exclusively^40^. Parameters used for defining and measuring disclosure also varied, with the most common parameters outlined as participant disclosure of their use of CM within the last twelve months to a medical provider (n = 30)^31–33,36,38,40,45–50,52,54,57,62,65,67,68,70,71,73,81,82,84,85,87,88,95,100,115,116^. Others studies examined participants’ disclosure to a medical provider of their current CM use^35,74,77–79,83,98,109,111^, use within the last month^34,53,69,86^, use within the last 24 months^50,51^, had always/usually/sometimes/never disclosed^39,59,60,66,72,110^, had ever discussed their CM use with a conventional provider^37,43,64,75,76^, had partially or fully disclosed their CM use^56,114^, had disclosed when asked^41^, had discussed before use^92^, reported rates of disclosure per episode of use^89^, or how the patient felt about disclosing^80,112^. A number of papers did not explicitly define their parameters for measuring disclosure^42,44,55,58,61,63,90,91,93,94,96,97,99,101–108,113^.

**Figure 2:**
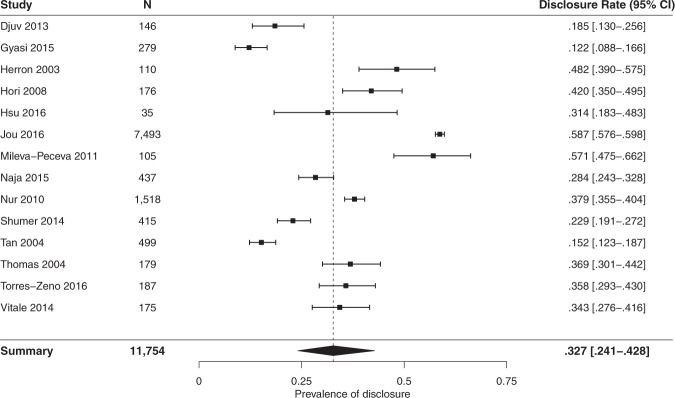
Meta-analysis results: disclosure rates for biologically-based complementary medicine. Results of meta-analysis assessing rates of disclosure of biologically-based complementary medicine use to medical providers.

The outcomes of the meta-analysis of the rate of disclosure of CM use by individuals using biologically-based CM is presented in Fig. 2. The measure of central tendency provided an overall disclosure rate of 33% (95% CI 24·1% to 42·8%, *I*^2^* = 98·6%*). Between the fourteen included studies, the lowest reported disclosure rate was 12% and the highest was 59%. Heterogeneity was assessed across the fourteen samples (Q-value 904.955, p < 0.001, I^2^ = 98.563). Although homogeneity was affected by the substantially larger sample size in Jou *et al*.’s 2016 study^54^, the paper was not excluded as it used a strong, internationally recognised dataset with very low risk of bias. The employment of a random effects model accounted for the impact of this study on homogeneity and its inclusion was not found to impact significantly on the measure of consistency within this model.

### Reasons for non-disclosure and disclosure

Twenty-five studies reported participant reasons for non-disclosure^36,37,42,54–57,59,67,76–79,83–85,90,92,98,105,107,110–113^, and four reported reasons for disclosure of CM use to medical providers^56,111–113^. The most commonly cited reasons patients gave for non-disclosure were fear of the provider’s disapproval^36,42,54–56,67,76–78,83–85,90,92,105,107,110–113^, followed by the provider not asking^37,42,54–57,59,67,76–78,83,84,90,98,110–113^, the patient perceiving disclosure as unimportant^42,54–57,59,67,76,78,79,84,85,90,92,98,105,107,110^, belief the physician would not have relevant knowledge of CM^36,42,54,56,67,76–78,107,113^, lack of time during consultation or forgetting^36,42,54,56,57,76,78,92,105^, belief that CM was safe and would not interfere with conventional treatment^42,78,83,85,111^, the patient not using CM regularly or at the time of consulting with the conventional provider^54,78,83,85^, and previous experiences of a negative response from conventional providers^54,84,90,112^. The most commonly cited reason for disclosure was that the provider asked about CM use^56,111,112^, followed by the patient expecting the provider to be supportive of their CM use^112,113^, believing disclosure was important for safety^56,113^, belief the provider would have relevant knowledge or advice about CM^56^, and belief that disclosing CM use may help other patients with the same condition^56^. Full details of reasons are shown in Table 5.

**Table 5:** Reasons for non-disclosure and disclosure.

	No. of studies	Studies reporting reason	Studies reporting as main reason^a^
**Reasons for non-disclosure**			
Patient was afraid of physician’s response or thought physician will disapprove	20	^36, 42, 54– 56, 67, 76– 78, 83– 85, 90, 92, 105, 107, 110– 113^	
Physician didn’t ask or wasn’t interested	19	^37, 42, 54– 57, 59, 67, 76– 78, 83, 84, 90, 98, 110– 113^	^54– 57, 77, 84^
Patient didn’t think it was important or necessary	18	^42, 54– 57, 59, 67, 76, 78, 79, 84, 85, 90, 92, 98, 105, 107, 110^	^59, 67, 76, 78, 79^
Didn’t think physician had relevant knowledge/wasn’t their business to know	10	^36, 42, 54, 56, 67, 76– 78, 107, 113^	^36^
No time/physician too busy/didn’t think about it/forgot	9	^36, 42, 54, 56, 57, 76, 78, 92, 105^	^42^
Thought CM was safe/wouldn’t interfere with treatment	4	^78, 83, 85, 111^	^83^
Was not using CM at the time/not using CM regularly/not attending a physician at the time	4	^54, 78, 83, 85^	^85^
Previous negative response or bad experience with disclosing	4	^54, 84, 90, 112^	
Patient had enough knowledge about CM	1	^42^	
Wanted to compare advice between conventional and CM practitioners	1	^113^	
Desire to protect cultural knowledge about CM	1	^113^	
Concerns physician will see patient’s CM use as detracting from their income	1	^113^	
**Reasons for disclosure**			
Physician asked	3	^56, 111, 112^	
Patient believed physician would be supportive	2	^112, 113^	
Patient believed it was important for safety reasons	2	^56, 113^	^56^
Patient believed physician would have relevant knowledge or advice about CM	1	^56^	
To help someone else with the same condition	1	^56^	

^a^Studies in which the corresponding reason was the reason most commonly reported by participants.

When participants were asked whether they thought disclosure was important, more than 67% agreed it was^36,63,68,80,110^. This percentage was highest (93%) among participants who were surveyed in CM clinics^68^, which was consistent with other studies reporting higher disclosure rates among users of practitioner-provided CM compared with self-administered CM^50,51,81,89^. Conversely, one study found lower disclosure rates among those using practitioner-provided CM, specifically where participants were consulting a CM practitioner and a medical provider for the same condition^65^.

### Impact of provider response on decisions to disclose

In a qualitative analysis, Shelley *et al*. found patients’ perceptions of how their medical provider might respond to their CM use was an important factor in the decision of whether or not to disclose^112^. A perception of the medical provider as accepting and non-judgemental encouraged disclosure while fear of a negative response from their medical provider led to non-disclosure^112^. One paper reported 59% of participants wanted to discuss CM with their medical provider (despite only 49% having done so), and 37% of non-disclosers wished it were easier to have such discussions^35^. In another study, the percentage of participants who wanted to discuss CM with their provider represented a substantial majority at 82% (despite only 60% having done so)^61^.

When the actual response of the provider to disclosure of CM use was explored by researchers, negative or discouraging responses were reported by a minority of respondents representing less than 20% of disclosers^65,71,77,85,105^, or were not reported at all^111^. However, in five papers positive or encouraging responses to disclosure of CM use by a medical doctor were reported by a substantial proportion of respondents representing 32–91% of disclosers^63,65,77,79,85,105^. Neutral responses from medical providers were also common, reported by 8–32% of disclosers in three studies^77,85,111^.

## Discussion

This review and meta-analysis provides a detailed overview and update of CM use disclosure to medical providers. Regarding the update to the 2004 paper^20^ afforded by this review, a substantially larger volume of literature reporting on CM disclosure was identified in our search, suggesting an increase in researcher interest in this aspect of patient-provider communication. Our analysis reveals little discernible improvement to disclosure rates over the last thirteen years. Consistent with the findings of the previous review, we found reports of disclosure vary widely. However, our additional meta-analysis on selected papers shows approximately two in three CM users do not disclose their CM use to medical providers. In view of the potential risks associated with unmanaged concomitant use of conventional and complementary medicine^11,14^, the value of increasing this rate of disclosure is accentuated.

Furthermore, our narrative review identified three distinct yet interrelated findings relating to patient-practitioner communication. Firstly, disclosure of CM use to medical providers is influenced by the nature of providers’ communication style; secondly, perceived provider knowledge of CM use is a barrier to discussions of CM use in clinical consultation; and thirdly, such discussions and subsequent disclosure of CM use may be facilitated by direct inquiry about CM use by providers. We consider this in the context of contemporary person-centred health care models.

Communication style was a repeated factor affecting disclosure rates in this review; disclosure of CM use was found to be encouraged by patient perceptions of acceptance and non-judgement from medical providers^112^, and inhibited by patient fears or previous experiences of discouraging responses from providers^36,42,54–56,67,76–78,83–85,90,92,105,107,110–113^. In practice, negative responses from medical providers appear to represent a deviation from the more commonly positive or neutral responses noted by participants of the reviewed studies as well as others^117,118^. However, such fears and subsequent non-disclosure of CM use could potentially be addressed by medical providers through communication with patients about CM in a direct, supportive, non-judgemental manner to build trust and communicative success^119^.

The reviewed literature shows patient perceptions of medical providers as lacking relevant knowledge about CM is a notable reason for non-disclosure. While examination of provider attitudes was not within the scope of this review, three reviewed papers included an assessment of medical providers’ attitudes toward discussing CM and identified lack of CM knowledge as a cause of providers’ reluctance to initiate such discussions^76,111,112^. Providers’ own perceived lack of CM knowledge as an obstacle to patient-provider CM communication also reflects other research examining provider perspectives on CM^120,121^. While the inclusion of CM in medical school curricula does occur in some countries (e.g. the US^122^, Canada^123^, UK^124^, Germany^125^, and Switzerland^126^), and is of interest to medical students^127,128^, this level of CM learning appears insufficient to equip medical providers with the confidence to address patient CM queries^120,121^. Furthermore, the depth and scope of CM knowledge to be realistically encouraged amongst medical providers has been contested^124,125^ and may be best facilitated on a case by case basis taking into account the circumstances of both provider and patient involved. Ideally, regardless of the level of CM knowledge held, the medical provider should strive to facilitate overall coordination and continuity of care for patients covering all treatments and providers, including those of CM.

Our analyses suggest there may be a vital role for medical providers in facilitating patient preference by enquiring with patients about CM in order to help improve disclosure rates. Other studies show discussions in conventional medical settings about CM use are more commonly patient rather than provider initiated^118,129^, a pattern reflected in the findings of some papers in this review^35,68,76^. This pattern suggests provider initiation of such discussions may be an avenue for improving disclosure rates, which may be achieved by means such as standard inclusion of CM use inquiry in case-taking education for medical students, as is currently the case in Switzerland^130^. Indeed, examination of the impact on disclosure rates of specific questions related to dietary supplements found medical providers’ questioning more than doubled the rate of supplement use disclosure^131^. This communicative success may be facilitated through employment of person-centred approaches to clinical care, which encompass patient involvement in shared decision-making, provider empathy and recognition of patients’ values^119^, encouraging a shared responsibility for communication and subsequent discussion of CM use.

While this review provides insight which could be integral to improving patient care during concomitant use of CM and conventional medicine, it also reveals the complexities of patient-practitioner communication in contemporary clinical settings. Further research into the nature of prevailing communication patterns, including differences in disclosure behaviours between populations of different demographics, is needed. As research into disclosure becomes more nuanced and data collection more consistent (e.g. through development and use of standardised instruments), future research could examine changes in patterns of and influences on disclosure. Additionally, research exploring the relationship between communication and treatment outcomes is warranted to provide a richer, deeper understanding of the impact of patient care dynamics. Such understanding could arguably provide the scaffolding for robust, effective, efficient public health policy and practice guidelines.

### Limitations of this review

The findings from our review need to be considered within the context of certain limitations. The varied nature and lack of a consistent international definition of CM lend a high degree of heterogeneity to the collection of studies appraised^132^. Likewise, while the wide variation in disclosure rates is likely to be partially due to confounding factors relating to differences among target populations (e.g. age, gender), settings (e.g. hospital, community clinics), geographical location (e.g. country/region), and sample sizes, the absence of a standard, validated tool for measuring disclosure also impacts the analysis and reporting on disclosure rates. The heterogeneity produced by these limitations reduced the number of papers suitable for meta-analysis and prevented a more robust, fixed-model meta-analysis on this topic, as well as prohibiting meta-analyses of CM categories other than biologically-based CM due to insufficient data. Additionally, identifying a comprehensive selection of studies to review was difficult due to disclosure frequently being reported as a secondary outcome and thus not being mentioned in the paper’s title, abstract or keywords. However, these limitations have been minimised where possible by following systematic review best practice, and while remaining mindful of the limitations of our review, the importance of the findings presented here for contemporary healthcare practice and provision should not be underestimated.

## Conclusion

The rate of disclosure regarding CM use to medical providers remains low and it appears that disclosure is still a major challenge facing health care providers. This review, alongside previous research, suggests that patient decision-making regarding disclosure and non-disclosure of CM use to a medical provider is impacted by the nature of patient-provider communication during consultation and perceptions of provider knowledge of CM. The initiation of conversations about CM with patients and provision of consultations characterised by person-centred, collaborative communication by medical providers may contribute towards increased disclosure rates and mitigate against the potential direct and indirect risks of un-coordinated concurrent CM and conventional medical care. This is a topic which should be treated with gravity; it is central to wider patient management and care in contemporary clinical settings, particularly for primary care providers acting as gatekeeper in their patients’ care.

## Supplementary information

Supplementary Methods S1: Systematic Review Protocol
