# Supplementary material for: Disclosure of complementary medicine use to medical providers: a systematic review and meta-analysis
Source: Sci Rep. 2019 Feb 7;9:1573. doi: 10.1038/s41598-018-38279-8 (PMC6367405; doi:10.1038/s41598-018-38279-8)
Supplement: Supplementary file 1 — Supplementary Methods S1: Systematic Review Protocol [file 41598_2018_38279_MOESM1_ESM.pdf]

Supplementary Methods S1: Systematic Review Protocol

Disclosure of complementary medicine use to medical providers: Protocol for an update, systematic review and meta-analysis

REVIEW PROTOCOL

Review Topic: Rates of disclosure and non-disclosure of complementary medicine (CM) use by patients to conventional health care providers: an update on Robinson & McGrail 2004.

Protocol Author:

Hope Foley (PhD Candidate)

Corresponding Author:

Hope Foley
PhD Candidate (Public Health)
Faculty of Health, University of Technology Sydney, Ultimo, Australia
hope.m.foley@student.uts.edu.au

Review Authors:

Hope Foley[1], Dr Amie Steel[1], Dr Holger Cramer[1,2], Dr Jon Wardle[1], Prof Jon Adams[1]

Affiliations:

[1]Faculty of Health, University of Technology Sydney, 15 Broadway, Ultimo, NSW 2007, Australia

[2]Department of Internal and Integrative Medicine, Kliniken Essen-Mitte, Faculty of Medicine, University of Duisburg-Essen, 45141 Essen, Germany

Contributions:

Concept, design and methodology – HF and AS. Literature search and study selection – HF and AS. Data analysis – HF, HC and AS. Interpretation of findings – HF, AS, HC, JA and JW. Initial draft of manuscript – HF. Authoring, editing and proofing of manuscript – HF, AS, JA, HC and JW.

Support:

Hope Foley is supported by an Australian Government Research Training Program Scholarship. Distinguished Professor Jon Adams is supported by an ARC Professorial Future Fellowship. Dr Jon Wardle is supported by a NHMRC Translating Research into Practice Fellowship. No funding sources played any role in the concept, conduct or reporting of this research.

Rationale:

Concomitant use of CM and conventional medicines can carry a variety of risks and benefits. Effective communication between patients and providers is essential in order to ensure risks are minimised and benefits are optimised. Previous research from over a decade ago has shown that disclosure rates of CM use to conventional medical providers can vary widely and are often much lower than is desirable. The reasons patients give for not disclosing may offer insights into how disclosure rates might be improved. In order to identify whether or not disclosure rates are still lower than desired, and how communication about CM use between patients and providers might be optimised, a review of the current literature should be undertaken as an update on previous reviews of the topic.

Research Question:

To what extent do users of CM disclose this use to conventional medical providers, and what are their reasons for disclosing or not disclosing?

Objectives:

- To provide an update on the review by Robinson & McGrail (2004)
- To assess rates of disclosure of CM use to conventional medical providers
- To assess reasons for disclosing and not disclosing CM use to conventional medical providers
- To explore what else is currently known about disclosure of CM use to conventional medical providers

Search Strategy: Updated from Robinson & McGrail's (2004) methodology (initial search on CM and individual therapies, then search within for terms related to disclosure). Individual therapies for this update were chosen on the basis of modalities identified as prevalent/common in 2012 review by Frass et al.

Date search: Jan 2003-Dec 2016 (update from last review conducted on publications up to and including 2002)

Databases: AMED, CINAHL, MEDLINE with full text, PsycINFO.

Search terms: Complementary medicine; Complementary therapies; Alternative medicine; Alternative therapies; Natural medicine; Natural therapies; Acupuncture; Aromatherapy; Ayurveda; Chiropractic; Herbal Medicine; Herbalism/Herbalist; Phytotherapy/Phytotherapist; Homeopath/Homeopathy; Hypnosis/Hypnotherapy; Massage; Naturopath/Naturopathy; Nutrition/Nutritionist; Diet therapy; Vitamin therapy; Supplement; Osteopathy; Reflexology; Traditional Chinese medicine; Yoga; Disclosure;

Communication; Patient use; Reasons for use; Medical practitioner; General practitioner; Health care provider; Primary care provider; Physician.

Table 1. Search strategy

**Protocol title**	**Disclosure of complementary medicine use to medical providers: An update and systematic review**	
**Date**	Jan 2003 – Dec 2016	
**Database**	**Search String**	**Expanders**
**AMED** *EBSCOhost* (amed)<br>**CINAHL** *EBSCOhost* (cin20)<br>**MEDLINE with full text** *EBSCOhost* (mnh)<br>**PsycINFO** *EBSCOhost* (psyh)	**S1** (complementary medicine OR complementary therap* OR alternative medicine OR alternative therap* OR natural medicine OR natural therap* OR acupunctur* OR aromatherap* OR ayurved* OR chiropract* OR herbal* OR phytotherap* OR homeopath* OR hypnosis OR hypnotherap* OR massage OR naturopath* OR nutrition* OR diet therap* OR vitamin therapy OR supplement OR osteopath* OR reflexolog* OR traditional Chinese medicine OR yoga)<br>**AND S2** (disclos* OR communicat* OR patient use OR reasons for use OR discuss*)<br>**AND S3** (medical practi* OR general practi* OR health care provider OR primary care provider OR physician)	Apply related words,<br>Apply equivalent subjects.

Manual search to be undertaken of reference lists from reviews identified during search, of reference lists from papers selected for review, and according to authors' expertise in topic.

Inclusion Criteria:

1. Original, peer-reviewed, observational research published January 2003 to December 2016
2. Participants include users of both CM and conventional medicine.
3. Outcomes report rates of disclosure/non-disclosure and/or reasons for disclosure/non-disclosure of CM use to conventional health/medical practitioners.
4. CM defined as any service, product or practice outside of conventional/dominant medical system, whether self-prescribed or accessed through CM (non-conventional) practitioner.
5. Sample can be reasonably described as comprising members of the general population.
6. Any language.

Exclusion Criteria:

1. Experimental study designs.
2. Sample cannot be reasonably described as comprising members of the general population (e.g. disease-specific population).

Additional Eligibility Criteria for Meta-Analysis:

With respect to homogeneity, additional criteria will be applied to identify those papers suitable for meta-analysis.

1. Participants comprise consistent population (e.g. adults).
2. Disclosure rate well-defined and consistent between included studies.
3. Definition of CM or type of CM used consistent between included studies.
4. Acceptable score in risk of bias assessment.

Data Management:

Citations to be managed using EndNote (Clarivate Analytics) citation management software. A copy of the library is to be archived after each step of the selection process.

Selection Process:

Citations will be filtered first by assessing the paper's title, then by assessing abstract, in order to identify which papers require reading by full-text. Citations will be retained at each step if it is conceivable that the paper may meet eligibility criteria. Full text articles will then be screened against all inclusion/exclusion criteria. Selection of studies for meta-analysis will be undertaken after data extraction to identify those with sufficient homogeneity.

The filtering process will be undertaken by HF and overseen by AS, with a selected sample of eligible studies to be reviewed at each stage of screening. Discrepancies in opinion regarding which citations should be retained and which should be excluded will be resolved through discussion until consensus is reached.

Data Extraction:

A customised form will be used to systematically extract data from each retained full-text paper. This will include study characteristics (year, study design, location, setting, population, sample, funding sources) and details of disclosure/non-disclosure (rates, reasons), as well as space for additional disclosure-related data identified *a posteriori*.

Data extraction will be performed by HF and overseen by AS. Any data which is potentially relevant to the research question but does not explicitly fit within pre-defined variables will be discussed until consensus is reached in regards to its inclusion.

Outcomes:

1. Rates of disclosure of CM use to conventional medical providers.
2. Reasons for disclosure and/or non-disclosure of CM use to conventional medical providers.
3. Additional disclosure-related data identified *a posteriori*.

Critical Appraisal:

Hoy et al.'s tool for prevalence studies will be used to assess quality of reporting in included studies and risk of bias in regards to sample selection, non-response bias, measurement bias and analysis bias. This will be applied during selection of studies for meta-analysis. Studies which score poorly overall in the appraisal, or which demonstrate risk of bias regarding sample selection or calculation of disclosure rates will be excluded from meta-analysis.

Data Synthesis:

Narrative synthesis will be undertaken using heuristic appraisal of extracted data in order to describe prevalence and parameters of disclosure. Reasons for disclosure and non-disclosure will be coded and collated. Additional data identified *a posteriori* will be synthesised in the manner deemed most appropriate (dependent on the nature of the data) once extraction has been completed.

Contingent on sufficient homogeneity, meta-analysis will be conducted using Comprehensive Meta-Analysis V3 software (Biostat Inc. 2017). The principal summary measure will be disclosure of CM use to conventional medical providers, using events (number of disclosers) and subset sample size (number of CM users) to determine event rates.

Start Date:

February 2017.

Expected Completion Date:

August 2017.

[This protocol has been developed in accordance with PRISMA-P and MOOSE guidelines.]
